# Effectiveness and safety of abobotulinumtoxinA in pediatric lower limb spasticity: A phase IV, prospective, observational, multicenter study

**DOI:** 10.1111/dmcn.16428

**Published:** 2025-07-31

**Authors:** Mark E. Gormley, Edward Dabrowski, Mauricio R. Delgado, Ann Tilton, Asare Christian, Sarah Helen Evans, Anne‐Sophie Grandoulier, Jumaah Goldberg

**Affiliations:** ^1^ Gillette Children's Specialty Healthcare, St. Paul MN USA; ^2^ Oakland University School of Medicine & Beaumont Hospital Grosse Point MI USA; ^3^ University of Texas Southwestern Medical Center and Scottish Rite for Children Dallas TX USA; ^4^ LSUHSC and Children's Hospital of New Orleans New Orleans LA USA; ^5^ Good Shepherd Rehabilitation Hospital Allentown PA USA; ^6^ Children's Hospital of Philadelphia Philadelphia PA USA; ^7^ Ipsen, Boulogne‐Billancourt France; ^8^ Formerly of Ipsen Cambridge MA USA

## Abstract

**Aim:**

To assess the longitudinal attainment of patient‐centered, function‐related Goal Attainment Scaling Total (GAS T)‐score after repeated abobotulinumtoxinA (AboBoNT‐A) injections over a period of up to 30 months and up to 10 cycles.

**Method:**

In this prospective observational study, the investigators' clinical practices recruited patients aged 2 to 17 years with pediatric lower limb spasticity (PLLS). GAS T‐scores were assessed for each injection, and goals could be redefined at each visit; scores of 50 reflected goal achievement. Adverse events were recorded.

**Results:**

Of 210 patients in the effectiveness population, 171 had cerebral palsy and 163 were previously treated with a botulinum neurotoxin. Available Gross Motor Function Classification System levels showed that 31.3% (61 out of 195) of patients were non‐ambulatory. Mean (SD) cumulative GAS T‐score was 51.1 (9.3) across the study duration; 75.2% achieved their primary goals. GAS T‐scores were comparable between BoNT‐naïve and previously treated patients and between age groups, but higher in the ambulatory than the non‐ambulatory subgroup. Injection guidance techniques were used in more than 70% of patients in cycles 1 to 6. Of 242 patients in the safety population, 102 reported 392 treatment‐emergent adverse events, including 15 reporting 35 treatment‐related adverse events. Adverse events were generally mild to moderate.

**Interpretation:**

Overall, goals were achieved as, or better than, expected in most patients. AboBoNT‐A was well tolerated, with a low incidence of treatment‐related adverse events. These results indicate that AboBoNT‐A is an effective treatment option, with a positive risk–benefit profile, for patients with PLLS across disability levels.

## Graphical abstract

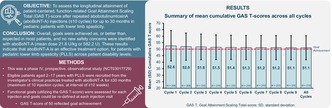

**Abbreviations**
AboBoNT‐A: abobotulinumtoxinA
GAS T: Goal Attainment Scaling Total
onaBoNT‐A: onabotulinumtoxinA
PedsQL: Pediatric Quality of Life Inventory
PLLS: pediatric lower limb spasticity
SAE: serious adverse event
TEAE: treatment‐emergent adverse event
USPI: US Prescribing Information

**What this paper adds**

- Treatment of spasticity with abobotulinumtoxinA improved patients' ability to achieve treatment goals.
- Ambulatory patients with cerebral palsy had a higher goal achievement rate than non‐ambulatory patients.
- Treatment with abobotulinumtoxinA was well tolerated, with a low incidence of adverse events.
- Serious adverse events were more frequent in non‐ambulatory than ambulatory patients.

Spasticity, characterized by a velocity‐dependent increase in muscle tone with exaggerated stretch reflexes, is a manifestation of the upper motor neuron syndrome that is caused by central nervous system lesions.^1^, ^2^ The extent and locations of lesions, rather than the cause of the lesions, are the main drivers of the diverse clinical presentations observed in spasticity.^3^, ^4^, ^5^ When treating pediatric lower limb spasticity (PLLS), regardless of presentation and muscles affected, therapeutic goals are to reduce muscle tone; improve strength, mobility, and coordination; minimize or delay development of contractures and limb deformity; and improve patients' quality of life.^6^, ^7^

Currently, most patients are managed with a combination of modalities, including physical, pharmacological, and surgical. Physical modalities (e.g. physical therapy, orthoses, and serial casting) are used independently or in combination with pharmacological treatments. Pharmacological treatments include botulinum neurotoxin type A (BoNT‐A), which targets specific muscles to treat focal and segmental spasticity, and systemic oral medications, which provide generalized anti‐spastic effects that are not muscle‐specific.^6^ Surgical management, because it is irreversible, is often reserved for children who are unresponsive to pharmacological treatment and for older children whose gait has sufficiently matured, to avoid repeated surgeries. Nonetheless, many of the currently approved pharmacological treatments for pediatric spasticity have significant limitations, and many are not approved for use in younger children.^6^, ^8^

BoNT‐A has been included in clinical guidance for the management of spasticity since 2000.^6^, ^7^, ^9^ AbobotulinumtoxinA (aboBoNT‐A) was approved for the treatment of PLLS in the USA in 2016. It is an important treatment intervention to help alleviate symptoms, improve function, delay or prevent the development of contractures, and support the achievement of motor milestones.^6^, ^9^, ^10^, ^11^ In view of the wide range of presentations and patient needs, the treatment of spasticity needs to be individualized, with an emphasis on patient‐centered goal setting and assessment as a clinical outcome.^7^

Goal attainment scaling (GAS) is an established patient‐centered method for defining individualized and quantifiable goals to improve function and participation.^12^, ^13^ GAS is a robust measure of differences between treatment groups and changes over time.^12^, ^14^, ^15^ Since goals are more likely to be achieved if patients are involved in setting them, GAS can be used to assess efficacy of treatments and is a sensitive marker of patient‐perceived improvement.^12^, ^16^ A single aggregated Total (T)‐score enables GAS scores to be standardized to indicate goals not achieved (<50), achieved (50), or overachieved (>50).^12^ To our knowledge, the rate of attainment of patient‐centered and function‐related goals after repeated aboBoNT‐A injections has not been collected in pediatric patients with lower limb spasticity in real‐world clinical practice. This gap is particularly evident for non‐ambulatory patients (Gross Motor Function Classification System [GMFCS] levels IV–V), who represent a significant subset of the population of patients with cerebral palsy (CP) yet are underrepresented in published literature.^17^

The primary objective of this study was to assess the longitudinal attainment of patient‐centered and function‐related goals (using the cumulative GAS T‐score) following repeated aboBoNT‐A injections and real‐world spasticity management up to 30 months and no more than 10 injection cycles. Long‐term safety was also assessed.

## METHOD

### Study design and participants

This was a phase IV, prospective, observational, multicenter study conducted at 27 US centers, each with at least one patient enrolled. Eligible patients aged 2 to 17 years with a primary diagnosis of PLLS were recruited from the investigators' clinical practices from 10th February 2017 to 30th May 2018. This study was designed to reflect real‐world clinical practice in the use of aboBoNT‐A. Treatment decisions were made before, and independently of, study enrollment. Patients could have been previously untreated (BoNT‐naïve) or treated with any formulation of BoNT‐A (including aboBoNT‐A), before study enrollment. Exclusion criteria included known resistance or lack of therapeutic response, as well as serious safety issues with previous BoNT chemodenervation, or concomitant chemodenervation with other BoNT.

Patients were followed up to 30 months and no more than 10 injection cycles at intervals of at least 12 weeks. AboBoNT‐A injection doses, intervals, number of injection points, and muscles injected were in accordance with the US Prescribing Information (USPI)^10^ and the physicians' clinical practices. AboBoNT‐A was indicated for lower limb spasticity in patients aged 2 years and older with the recommended dosing of 15 units (U)/kg for injections in one leg, or 30 U/kg for both legs, with a maximum dose of 1000 U, whichever was lower.

This study (NCT03017729) was conducted with appropriate institutional review board (Akron Children's Hospital, BRANY, Children's Hospital of Wisconsin, Cleveland Clinic, Good Shepherd Rehabilitation, University of Minnesota, University of Missouri‐Columbia, UT Southwestern Medical Center, Washington University St. Louis, and WCG) approval and in compliance with the Declaration of Helsinki and Good Pharmacoepidemiology Practice guidelines. Written informed consent was obtained from parent(s)/guardian(s), and assent from children when applicable, before enrollment. Patients and/or parents/caregivers, in consultation with investigators, identified primary and secondary functional goals (Table S1). Goals were assessed at the end of each injection cycle and could be redefined at each injection visit. Progress towards individual therapy goals was measured using a cumulative GAS T‐score.

Before treatment, goals were identified and defined on the basis of a range of possible outcomes for each goal area. The attainment levels for each goal were then combined in a single aggregated T‐score using a formula that accounts for variable numbers of goals and intercorrelation of goal areas to transform GAS into a standardized T‐score with a mean of 50. The GAS T‐score was derived from the Goal Attainment Scale, which is a 5‐point Likert scale ranging from −2 (much less than expected) to +2 (much more than expected) and quantifies the outcome in a single aggregated goal attainment score. A GAS T‐score of 50 represents goals achieved as expected, less than 50 less well than expected, and greater than 50 better than expected.^12^ Other outcomes, collected at each visit, included Pediatric Quality of Life Inventory (PedsQL), PedsQL CP, and patient‐ and caregiver‐reported outcomes (i.e. duration of treatment effect). Median time since the last injection (before study entrance) was obtained from the patients' records.

Adverse events were recorded at each visit, coded using MedDRA version 2.0, and assessed for seriousness and causality. Special situations were collected in the ‘Safety Report Form’ in the electronic case report form and included any incidence of ancillary use (i.e. injections with no formal dosing guidance), medication errors, occupational exposure, abuse, misuse, or lack of therapeutic efficacy while using the study drug. Post hoc analyses explored seizure occurrence in relation to study treatment.

### Statistical analyses

A total of 237 participants were planned for enrollment assuming a 20% dropout rate during the follow‐up period. Determination of the sample size was based on the cumulative GAS T‐score across the entire study, with the aim of estimating it with a precision of ±1.5 points (assuming a standard deviation [SD] of 10.5 and a two‐sided 95% confidence interval [CI]).

Safety analyses were performed in all patients who were injected at least once with aboBoNT‐A during the study (treated/safety population), including by age group at enrollment. Effectiveness was assessed in all patients who were injected at least once with aboBoNT‐A during the study and underwent at least one assessment of goal attainment (effectiveness population).

As this was an observational study, all results are descriptive. Summary statistics include number of available observations (*n*), number of missing values (missing), arithmetic mean, 95% CI of the mean, SD, median, first and third quartile (Q1, Q3), and the range (minimum, maximum). For categorical/discrete variables, the absolute and relative (percentage) numbers are presented, including Agresti–Coull approximate 95% CIs for binomial proportions. No imputations were performed for the effectiveness endpoints and missing/incomplete data were left as they were recorded.

All analyses were conducted using SAS version 9.4 or higher (SAS Institute Inc., Cary, NC, USA).

## RESULTS

Of 243 patients enrolled, 242 were included in the treated/safety population and 210 in the effectiveness population (Figure S1). Of the effectiveness population, 143 (68.1%) were aged 2 to 9 years, 135 (64.3%) were male, and 163 (77.6%) had previously been treated with BoNT‐A (Table 1). The median time since PLLS onset was 69.6 months (range 0.5–202.1), with the most frequent cause leading to PLLS being CP (82.6%). Available GMFCS levels showed that 31.3% (*n =* 61 out of 195) of the patients were non‐ambulatory (GMFCS levels IV–V).

**TABLE 1:** Baseline demographics and characteristics (effectiveness population).

	BoNT‐naïve	Previously treated	Total population
	(*n =* 47)	(*n =* 163)	(*n* = 210)
Sex, *n* (%)	47	163	210
Male	32 (68.1)	103 (63.2)	135 (64.3)
Female	15 (31.9)	60 (36.8)	75 (35.7)
Age at enrollment, *n*	47	163	210
Mean (SD), years:months	6:2 (4:2)	8:0 (4:2)	7:7 (4:2)
Minimum, maximum	2, 17	2, 16	2, 17
Age category at enrollment, *n* (%)	47	163	210
2–9 years	37 (78.7)	106 (65.0)	143 (68.1)
10–17 years	10 (21.3)	57 (35.0)	67 (31.9)
Weight (kg), *n*	45	155	200
Mean (SD)	26.6 (18.7)	28.0 (15.5)	27.7 (16.2)
Minimum, maximum	9.0, 84.7	10.4, 84.9	9.0, 84.9
Concomitant non‐drug therapy (≥10% of patients), *n* (%)			
Physical therapy	—	—	134 (63.8)
Cast application	—	—	99 (47.1)
Occupational therapy	—	—	89 (42.4)
Speech rehabilitation	—	—	35 (16.7)
Type of acquired brain injury, *n* (%)			207
Cerebral palsy	—	—	171 (82.6)
Stroke	—	—	10 (4.8)
Brain trauma	—	—	6 (2.9)
Spinal cord injury	—	—	5 (2.4)
Other	—	—	15 (7.2)
Motor type, *n* (%)			
Unilateral	15 (31.9)	38 (23.3)	53 (25.2)
Bilateral	32 (68.1)	125 (76.7)	157 (74.8)
Most affected leg, *n* (%)			
Right	25 (78.1)	81 (64.8)	106 (67.5)
Left	7 (21.9)	44 (35.2)	51 (32.5)
GMFCS level, *n* (%)	38	157	195
I	9 (23.7)	28 (17.8)	37 (19.0)
II	12 (31.6)	52 (33.1)	64 (32.8)
III	6 (15.8)	27 (17.2)	33 (16.9)
IV	4 (10.5)	22 (14.0)	26 (13.3)
V	7 (18.4)	28 (17.8)	35 (17.9)

Abbreviations: BoNT, botulinum neurotoxin; GMFCS, Gross Motor Function Classification System; SD, standard deviation.

The typical treatment interval (based on the number of patients previously treated with BoNT‐A) was longer than 12 weeks in 57.2% of patients, 12 weeks exactly in 39.1%, and shorter than 12 weeks in 3.6%. In patients whose usual treatment interval was longer than 12 weeks, the reason for the interval duration was patient need in 73.1%. Overall, the median time since last injection at study entry was 21.4 weeks, with 54.3% of patients receiving onabotulinumtoxinA (onaBoNT‐A), 42.6% receiving aboBoNT‐A, and 3.1% receiving incobotulinumtoxinA during their previous injection visit. The median time since last onaBoNT‐A injection was longer than for aboBoNT‐A (26.0 vs 18.3 weeks), with a mean lower limb total dose of 224.8 and 467.7 U for onaBoNT‐A and aboBoNT‐A respectively; however, the median duration of effect was notably longer for aboBoNT‐A than onaBoNT‐A (16.0 vs 12.0 weeks respectively).

Past or current significant medical and/or surgical history was reported in 85.2% of patients. The most common medical conditions were nervous system disorders (58.6%), which included seizures in 23.8% (*n =* 50 out of 210) of the effectiveness population and 33.5% (*n =* 81 out of 242) of the safety population. Additionally, surgical and medical procedures were reported in 41.9% of patients; congenital, familial, and genetic disorders in 33.3%; and gastrointestinal disorders in 25.2%.

Overall, 76.2% of patients received at least three aboBoNT‐A injection cycles, with a 22.7‐week median for the average duration of cycles per patient, consistent with the USPI.^10^ The median duration of cycle 1 was 20.1 weeks (range 11.1–152.0), decreasing to 14.3 weeks (range 12.1–17.7) at cycle 9. Across all cycles, the mean (SD) number of muscles injected ranged from 5.5 (2.9) to 7.0 (3.7), with the overall mean number of injection points ranging from 8.1 (2.7) to 9.9 (5.7) (Table 2). The percentage of patients with both lower and upper limb injections ranged from 18.4% to 42.9% across all cycles.

**TABLE 2:** AbobotulinumtoxinA injection patterns (effectiveness population).

	Cycle 1	Cycle 2	Cycle 3	Cycle 4	Cycle 5	Cycle 6	Cycle 7	Cycle 8	Cycle 9	Cycle 10
	(*n* = 210)	(*n* = 194)	(*n* = 160)	(*n* = 130)	(*n* = 98)	(*n* = 74)	(*n* = 49)	(*n* = 29)	(*n* = 18)	(*n* = 7)
Administration performed^a^, *n* (%)	210 (100.0)	194 (100.0)	160 (100.0)	130 (100.0)	98 (100.0)	74 (100.0)	49 (100.0)	29 (100.0)	18 (100.0)	7 (100.0)
Both lower and upper limb^a^	46 (21.9)	47 (24.2)	40 (25.0)	32 (24.6)	23 (23.5)	16 (21.6)	9 (18.4)	7 (24.1)	6 (33.3)	3 (42.9)
Lower limb only^a^	164 (78.1)	146 (75.3)	119 (74.4)	98 (75.4)	75 (76.5)	57 (77.0)	40 (81.6)	22 (75.9)	12 (66.7)	4 (57.1)
Upper limb only^a^	0	1 (0.5)	1 (0.6)	0	0	1 (1.4)	0	0	0	0
Number of muscles injected^a^										
Mean (SD)	6.0 (3.3)	6.0 (3.1)	6.2 (3.1)	6.1 (3.3)	6.2 (3.4)	5.7 (3.2)	5.7 (3.2)	5.7 (2.8)	5.5 (2.9)	7.0 (3.7)
Number of sites injected^a^										
Mean (SD)	9.7 (6.3)	9.6 (5.8)	9.6 (5.3)	9.9 (5.7)	9.5 (5.3)	9.2 (5.2)	9.0 (4.8)	8.3 (4.0)	8.1 (2.7)	8.4 (3.5)
Total injected dose in lower and upper limb^a^, *n* (%)	210 (100.0)	193 (99.5)	160 (100.0)	130 (100.0)	98 (100.0)	74 (100.0)	49 (100.0)	29 (100.0)	18 (100.0)	7 (100.0)
Mean (SD) total injection dose (U)^a^	540.3 (241.4)	552.1 (259.7)	578.4 (260.6)	589.5 (256.3)	610.8 (274.5)	650.0 (300.9)	611.2 (256.6)	681.2 (270.5)	709.2 (256.2)	814.3 (221.2)
Minimum, maximum total injection dose (U)	100, 1250	40, 1500	150, 1250	220, 1400	250, 1500	300, 1500	300, 1500	400, 1500	350, 1200	500, 1000

Abbreviations: SD, standard deviation; U, units.

^a^ On the basis of the number of injected patients.

Overall, the mean (SD) total body injected aboBoNT‐A dose was 21.8 (7.3) U/kg and was higher in the non‐ambulatory (GMFCS levels IV–V) than ambulatory (GMFCS levels I–III) subgroup (Table 3). Gastrocnemius muscle injections were the most commonly injected, with more than 80% of patients receiving medial or lateral head injections, with a mean (SD) total aboBoNT‐A injected dose of 4.3 (2.4) and 3.7 (2.2) U/kg respectively (Table 3). The mean total injected doses were notably lower in the non‐ambulatory than ambulatory subgroup for both the medial and lateral head injections. Ancillary lower limb injections were reported in 82.9% of patients compared with 27.1% for ancillary upper limb injections, with hamstrings (61.0%) and adductor magnus (26.7%) the most frequently injected muscles.

**TABLE 3:** AbobotulinumtoxinA administration in most common muscles (effectiveness population).

	Total	Ambulatory	Non‐ambulatory
	(*n* = 210)	(*n* = 134)	(*n* = 61)
Total body injected dose, *n* (%)	210 (100.0)	134 (100.0)	61 (100.0)
Total body injected dose (U/kg)			
Mean (SD)	21.8 (7.3)	20.2 (7.2)	25.8 (6.1)
Median (IQR)	22.5 (15.9, 27.0)	20.2 (14.6, 25.1)	26.7 (15.5, 24.8)
Total body injected dose (U)			
Mean (SD)	582.2 (247.6)	583.2 (244.4)	604.2 (251.8)
Median (IQR)	520.4 (400.0, 780.0)	521.5 (406.0, 750.0)	530.0 (430.0, 805.0)
Mean (SD) number of injection points	1.9 (1.1)	1.9 (1.2)	1.7 (0.8)
Gastrocnemius (medial head), *n* (%)	180 (85.7)	122 (91.0)	45 (73.8)
Total injected dose (U/kg)			
Mean (SD)	4.3 (2.4)	4.6 (2.6)	3.7 (2.2)
Median (IQR)	3.7 (2.6, 5.6)	4.1 (2.7, 6.0)	3.1 (2.3, 4.1)
Mean (SD) total injected dose (U)	112.7 (65.9)	126.4 (66.6)	78.8 (40.7)
Mean (SD) number of injection points	1.8 (0.9)	1.9 (1.0)	1.5 (0.8)
Gastrocnemius (lateral head), *n* (%)	173 (82.4)	119 (88.8)	41 (67.2)
Total injected dose (U/kg)			
Mean (SD)	3.7 (2.2)	4.0 (2.5)	2.9 (1.5)
Median (IQR)	3.1 (2.3, 4.6)	3.4 (2.4, 5.1)	2.5 (2.1, 3.2)
Mean (SD) total injected dose (U)	96.7 (59.1)	107.5 (58.3)	63.6 (34.9)
Mean (SD) number of injection points	1.6 (0.7)	1.7 (0.7)	1.4 (0.6)
Soleus, *n* (%)	131 (62.4)	91 (67.9)	31 (50.8)
Total injected dose (U/kg)			
Mean (SD)	3.1 (1.9)	3.2 (1.6)	3.0 (2.6)
Median (IQR)	2.7 (1.7, 4.0)	3.0 (2.0, 4.2)	2.3 (1.5, 3.7)
Mean (SD) total injected dose (U)	84.5 (56.9)	94.1 (57.6)	62.3 (45.9)
Mean (SD) number of injection points	1.5 (0.7)	1.6 (0.7)	1.3 (0.6)
Hamstrings, *n* (%)	128 (61.0)	74 (55.2)	44 (72.1)
Total injected dose (U/kg)			
Mean (SD)	6.4 (3.5)	6.4 (3.3)	6.9 (4.1)
Median (IQR)	5.5 (3.9, 7.7)	5.6 (3.9, 8.0)	5.8 (4.2, 8.4)
Mean (SD) total injected dose (U)	185.0 (120.2)	205.4 (124.3)	170.2 (106.6)
Mean (SD) number of injection points	2.7 (1.6)	2.8 (1.6)	2.6 (1.5)
Adductor magnus, *n* (%)	56 (26.7)	15 (11.2)	35 (57.4)
Total injected dose (U/kg)			
Mean (SD)	4.7 (3.9)	3.5 (2.1)	5.3 (4.6)
Median (IQR)	3.4 (2.3, 6.1)	3.2 (2.0, 4.9)	3.7 (2.6, 7.8)
Mean (SD) total injected dose (U)	90.8 (71.1)	72.3 (49.3)	103.3 (81.5)
Mean (SD) number of injection points	1.3 (0.5)	1.2 (0.4)	1.4 (0.5)
Adductor longus, *n* (%)	48 (22.9)	10 (7.5)	34 (55.7)
Total injected dose (U/kg)			
Mean (SD)	4.1 (2.9)	3.7 (1.6)	4.2 (3.2)
Median (IQR)	3.2 (2.4, 4.9)	3.4 (2.9, 3.9)	3.2 (2.6, 4.9)
Mean (SD) total injected dose (U)	84.0 (62.0)	85.2 (49.7)	87.3 (68.0)
Mean (SD) number of injection points	1.4 (0.6)	1.7 (0.8)	1.3 (0.5)

*Note*: Ambulatory status was defined as Gross Motor Function Classification System (GMFCS) levels I–III; non‐ambulatory status as GMFCS levels IV–V.

Abbreviations: IQR, interquartile range; SD, standard deviation; U, units.

For all aboBoNT‐A injections, 200 U/mL was the most frequently used dilution. Injection guidance techniques were used in more than 70% of patients in cycles 1 to 6; electrostimulation was most common (>50%). Sedation was more frequently used at early cycles (cycle 1: 53.8% vs 10: 14.3%), with benzodiazepine derivatives and halogenated hydrocarbons being most common.

Across all cycles, most patients reported concomitant non‐drug (81.4%) and drug (41.9%) therapies. The most common concomitant non‐drug therapies (defined as at least one occurrence) in the total population were physical modalities, which included physical/occupational therapy and cast application (Table 1). Despite being reported by most, only a fraction of patients used concomitant physical modalities for the duration of the study. The most common concomitant drug therapy was enteral anti‐spasticity medications (35.2%), specifically baclofen (34.3%).

**FIGURE 1:**
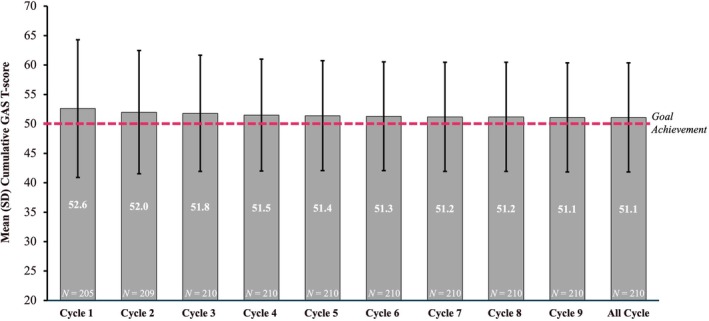
Summary of mean cumulative GAS T‐scores across all cycles (effectiveness population). Abbreviations: GAS T‐score, Goal Attainment Scaling Total‐score; SD, standard deviation.

**FIGURE 2:**
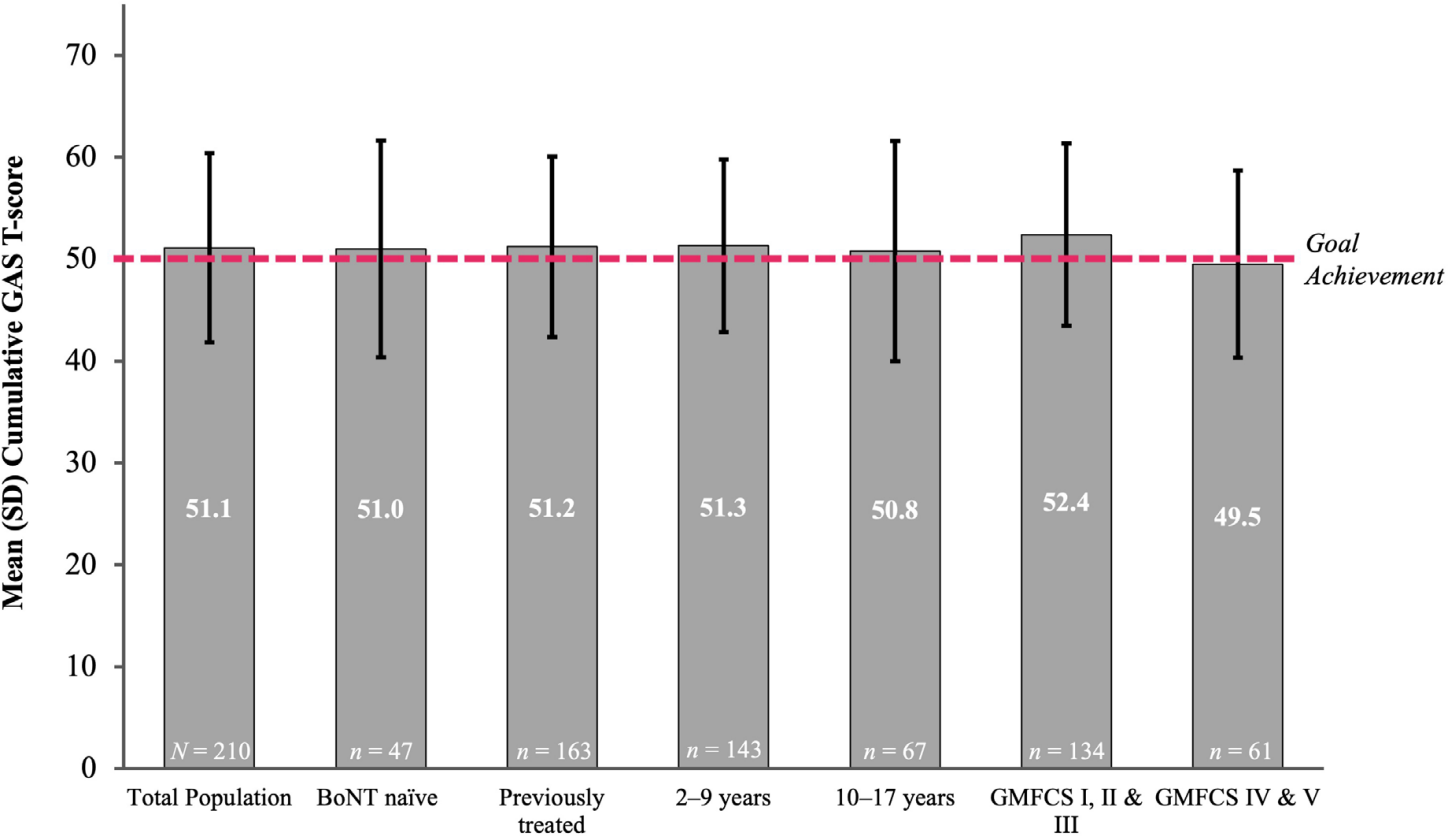
Mean cumulative GAS T‐scores by subgroup and overall (effectiveness population). Abbreviations: BoNT, botulinum neurotoxin; GAS T‐score, Goal Attainment Scaling Total‐score; GMFCS, Gross Motor Function Classification System; SD, standard deviation.

**FIGURE 3:**
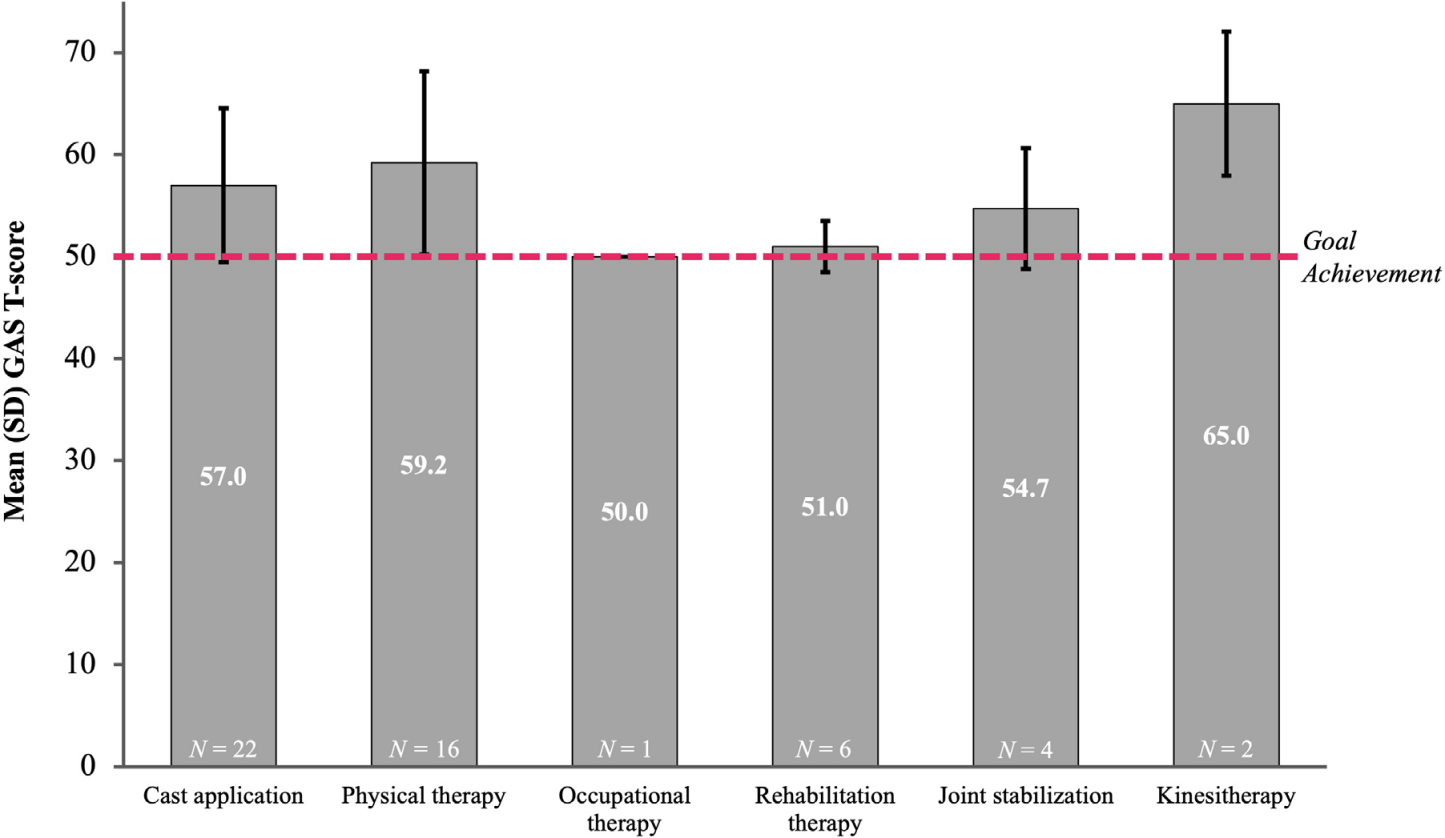
Mean GAS T‐scores at end of study by concomitant physical modalities (effectiveness population). Patients with only one non‐drug therapy. Abbreviations: GAS T‐score, Goal Attainment Scaling Total‐score; SD, standard deviation.

The overall mean (SD) cumulative GAS T‐score for the total population was 51.1 (9.3) (Figure 1). Mean cumulative GAS T‐scores indicated patients' ability to achieve their treatment goals across subgroups; GAS T‐scores were comparable between BoNT‐naïve and previously treated patients, as well as between the 2 to 9 years and 10 to 17 years age groups; however, ambulatory patients had a greater level of goal achievement than the non‐ambulatory patients (Figure 2). Patients who used cast application, physical or occupational concomitant therapies throughout the study achieved mean cumulative GAS T‐scores ranging from 50.0 (0.0) to 59.2 (8.97) (Figure 3). Overall, the mean cumulative GAS T‐scores were comparable across all the concomitant physical modalities that were used by the patients and reflected goal overachievement.

**FIGURE 4:**
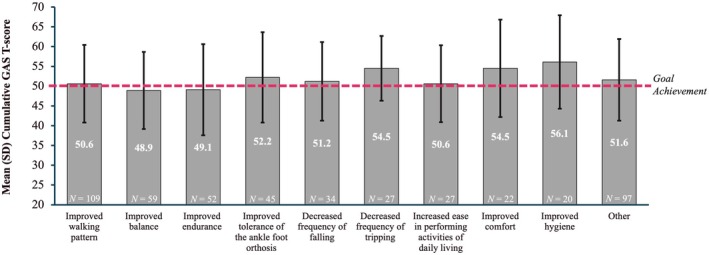
Cumulative GAS T‐scores across all cycles by goal area (effectiveness population). Abbreviations: GAS T‐score, Goal Attainment Scaling Total‐score; SD, standard deviation.

**FIGURE 5:**
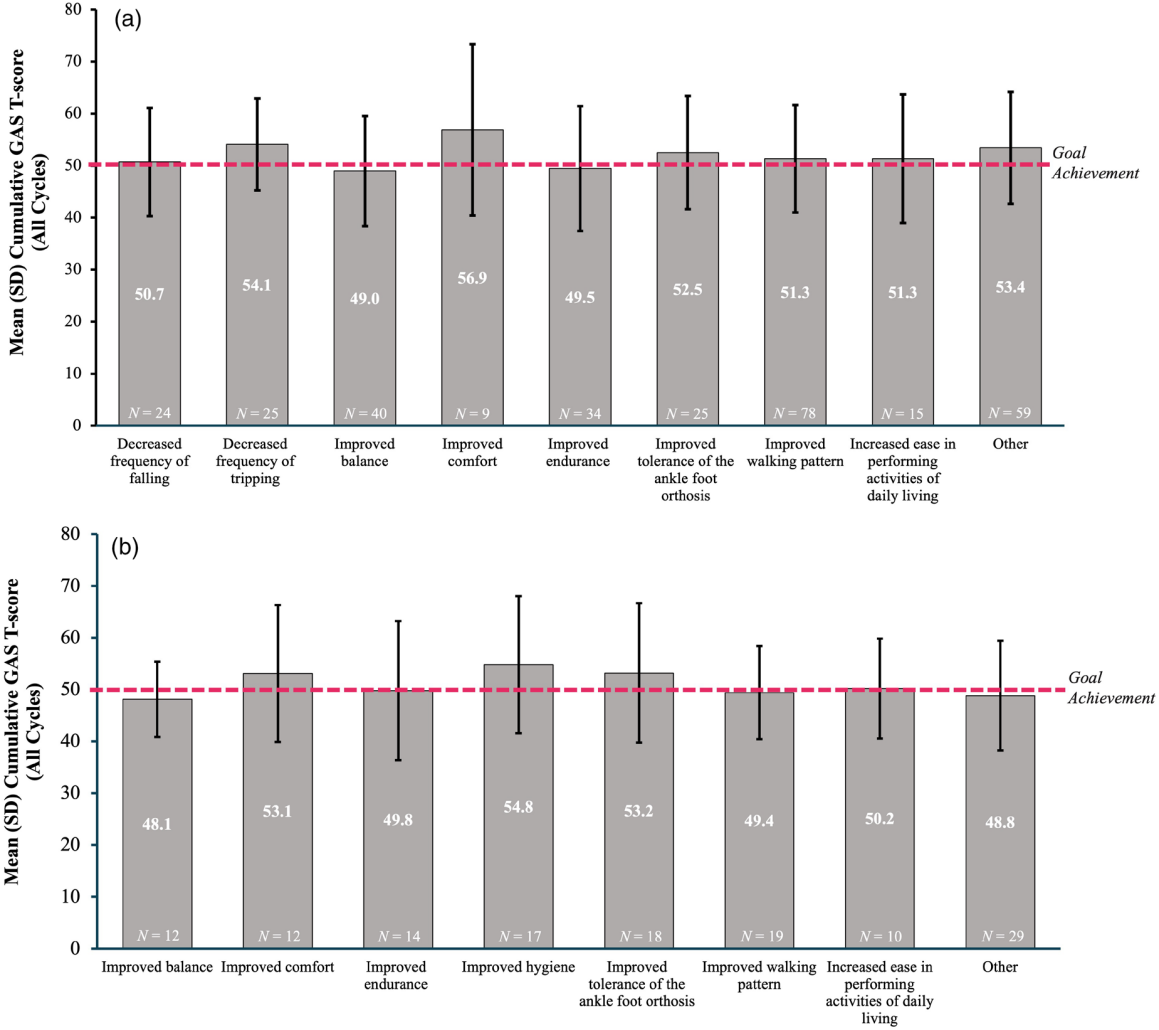
Cumulative GAS T‐scores across all cycles by goal area in (a) ambulatory and (b) non‐ambulatory subgroups (effectiveness population). Goal areas chosen by at least 20 patients overall. Ambulatory status was defined as GMFCS levels I–III; non‐ambulatory status as GMFCS levels IV–V. Note the goal of ‘improved walking pattern’ selected by seven patients in the GMFCS level V subgroup may reflect aims related to robotic‐assisted devices or pre‐walking/assisted stepping movements. Abbreviations: GAS T‐score, Goal Attainment Scaling Total‐score; GMFCS, Gross Motor Function Classification System; SD, standard deviation.

The most frequently selected goals throughout the study were improved walking pattern, improved balance, improved endurance, improved tolerance of the ankle–foot orthosis, decreased frequency of falling, decreased frequency of tripping, increased ease in performing activities of daily living, improved comfort, and improved hygiene (Table S2). For most of these, a cumulative GAS T‐score of greater than 50 across all cycles was observed, with slightly lower scores for the improved balance and improved endurance (Figure 4). A similar trend was seen in the cumulative GAS T‐score across all cycles in the ambulatory (Figure 5a) and non‐ambulatory (Figure 5b) subgroups.

At the end of cycle 1, 74.8% of patients achieved their GAS T primary goal, with more than 60% achieving it through the end of cycle 8, reflecting sustained goal attainment (Table S3). Consistent with these results, 75.2% of patients achieved GAS T primary goals overall. Achievement overall was reached if the number of achieved primary goals across all cycles exceeded the number of non‐achieved primary goals. If the two numbers were equal, the achievement status of the last cycle was considered the overall achievement status.

Patients' or parents' satisfaction with treatment, assessed using the PedsQL and PedsQL CP, was completed at each visit and was summarized as a total score as well as separately for parent and patient. Improvement in the PedsQL total score was observed at cycles 8 and 9, with a mean change from baseline of 5.50 (95% CI 0.51–10.49) and 7.73 (95% CI −0.20 to 15.67) respectively. Improvements in the PedsQL physical health summary score was observed at cycles 2 and 3, with a mean change of 6.41 (95% CI 1.59–11.23) and 7.91 (95% CI 2.71–13.11) respectively. Similar trends were observed for the parents' PedsQL total and physical health summary scores, but not for the patients' PedsQL total score, suggesting differences in the perception of the impact of PLLS on the quality of life between parents and patients. PedsQL CP scores improved in daily activities (from cycles 2 to 8), pain and hurt (cycle 2), and eating activities dimensions (cycles 2, 3, and 8).

Most patients aged 10 to 17 years (90.9%) and their caregivers (87.7%) reported being satisfied with previous BoNT‐A treatment (aboBoNT‐A or other variety), with all (100%) being satisfied or very satisfied with previous aboBoNT‐A specifically. The estimated median time to retreatment (duration of effect) by the caregiver ranged from 14.0 to 16.1 weeks from cycles 1 to 6 and was notably shorter than the actual median duration of injection cycles (22.7 weeks), consistent with the USPI.^10^ Most caregivers identified duration of effect (i.e. effects of treatment start wearing off) as their main rationale for retreatment.

In the safety population, 102 patients (42.1%) reported 392 treatment‐emergent adverse events (TEAEs), with most TEAEs being mild to moderate (Table 4). The incidence of TEAEs was comparable between age groups and ambulatory versus non‐ambulatory subgroups. The five most common TEAEs were seizures, upper respiratory tract infection, pyrexia, influenza, and falls (Table 4). No incidences of seizure were considered treatment‐related, including in patients with previous seizure history (*n =* 10 out of 81, 12.3%) and without previous seizure history (*n =* 6 out of 161, 3.7%). Of the six without previous seizures who experienced a seizure, five had a diagnosis of CP and were non‐ambulatory. Treatment‐related adverse events were reported in 15 patients (6.2%), totaling 35 events. The most common were asthenia (1.2% of patients), dysphagia (1.2%), muscular weakness (1.2%), pain in extremity (0.8%), and falls (0.8%).

**TABLE 4:** Summary of safety and tolerability (safety population).

	2–9 years	10–17 years	Ambulatory	Non‐ambulatory	Total
	(*n* = 158)	(*n* = 83)	(*n* = 149)	(*n* = 75)	(*n* = 242)
Any adverse event, *n* (%)	68 (43.0)	34 (41.0)	65 (43.6)	32 (42.7)	102 (42.1)
Severe	12 (7.6)	5 (6.0)	9 (6.0)	8 (10.7)	17 (7.0)
Moderate	36 (22.8)	18 (21.7)	31 (20.8)	19 (25.3)	54 (22.3)
Mild	53 (33.5)	28 (33.7)	56 (37.6)	24 (32.0)	81 (33.5)
Treatment‐related, *n* (%)	8 (5.1)	7 (8.4)	11 (7.4)	4 (5.3)	15 (6.2)
Not related, *n* (%)	65 (41.4)	32 (38.6)	62 (41.6)	30 (40.0)	97 (40.1)
SAE, *n* (%)	19 (12.0)	6 (7.2)	12 (8.1)	13 (17.3)	25 (10.3)
TEAE leading to study drug withdrawal, *n* (%)	0	2 (2.4)	0	1 (1.3)	2 (0.8)
TEAE leading to death, *n* (%)	0	0	0	0	0
Infections and infestations, *n* (%)	37 (23.4)	16 (19.3)	34 (22.8)	16 (21.3)	53 (21.9)
Upper respiratory tract infection	11 (7.0)	0	6 (4.0)	5 (6.7)	11 (4.5)
Influenza	6 (3.8)	2 (2.4)	5 (3.4)	5 (6.7)	8 (3.3)
Otitis media	6 (3.8)	0	4 (2.7)	2 (2.7)	6 (2.5)
Otitis media acute	5 (3.2)	1 (1.2)	5 (3.4)	1 (1.3)	6 (2.5)
Pharyngitis	3 (1.9)	2 (2.4)	4 (2.7)	1 (1.3)	5 (2.1)
Pneumonia	4 (2.5)	1 (1.2)	1 (0.7)	4 (5.3)	5 (2.1)
Sinusitis	3 (1.9)	2 (2.4)	5 (3.4)	0	5 (2.1)
Urinary tract infection	3 (1.9)	2 (2.4)	2 (1.3)	3 (4.0)	5 (2.1)
Pharyngitis streptococcal	3 (1.9)	1 (1.2)	4 (2.7)	0	4 (1.7)
Viral upper respiratory tract infection	2 (1.3)	2 (2.4)	4 (2.7)	0	4 (1.7)
Viral infection	3 (1.9)	0	1 (0.7)	2 (2.7)	3 (1.2)
Musculoskeletal and connective tissue disorders, *n* (%)	17 (10.8)	11 (13.3)	19 (12.8)	8 (10.7)	28 (11.6)
Arthralgia	2 (1.3)	4 (4.8)	4 (2.7)	2 (2.7)	6 (2.5)
Pain in extremity	4 (2.5)	1 (1.2)	5 (3.4)	0	5 (2.1)
Tendinous contracture	3 (1.9)	1 (1.2)	4 (2.7)	0	4 (1.7)
Foot deformity	3 (1.9)	0	3 (2.0)	0	3 (1.2)
Muscle spasms	2 (1.3)	1 (1.2)	1 (0.7)	2 (2.7)	3 (1.2)
Muscular weakness	2 (1.3)	1 (1.2)	1 (0.7)	2 (2.7)	3 (1.2)
Nervous system disorders, *n* (%)	22 (13.9)	6 (7.2)	17 (11.4)	10 (13.3)	28 (11.6)
Seizure	15 (9.5)	1 (1.2)	8 (5.4)	5 (6.7)	16 (6.6)
Status epilepticus	4 (2.5)	0	2 (1.3)	2 (2.7)	4 (1.7)
Headache	3 (1.9)	0	2 (1.3)	1 (1.3)	3 (1.2)
Injury, poisoning and procedural complications, *n* (%)	20 (12.7)	5 (6.0)	18 (12.1)	7 (9.3)	25 (10.3)
Fall	5 (3.2)	2 (2.4)	7 (4.7)	0	7 (2.9)
Joint dislocation	5 (3.2)	0	1 (0.7)	4 (5.3)	5 (2.1)
Foreign body	3 (1.9)	0	3 (2.0)	0	3 (1.2)
Procedural pain	2 (1.3)	1 (1.2)	2 (1.3)	1 (1.3)	3 (1.2)
General disorders and administration site conditions, *n* (%)	15 (9.5)	6 (7.2)	17 (11.4)	4 (5.3)	21 (8.7)
Pyrexia	9 (5.7)	1 (1.2)	9 (6.0)	1 (1.3)	10 (4.1)
Asthenia	1 (0.6)	2 (2.4)	1 (0.7)	2 (2.7)	3 (1.2)
Gait disturbance	2 (1.3)	1 (1.2)	3 (2.0)	0	3 (1.2)
Gastrointestinal disorders, *n* (%)	15 (9.5)	5 (6.0)	13 (8.7)	7 (9.3)	20 (8.3)
Constipation	5 (3.2)	1 (1.2)	2 (1.3)	4 (5.3)	6 (2.5)
Dysphagia	2 (1.3)	3 (3.6)	4 (2.7)	1 (1.3)	5 (2.1)
Vomiting	5 (3.2)	0	3 (2.0)	2 (2.7)	5 (2.1)
Respiratory, thoracic and mediastinal disorders, *n* (%)	11 (7.0)	8 (9.6)	10 (6.7)	8 (10.7)	19 (7.9)
Asthma	2 (1.3)	1 (1.2)	3 (2.0)	0	3 (1.2)
Psychiatric disorders, *n* (%)	10 (6.3)	2 (2.4)	10 (6.7)	1 (1.3)	12 (5.0)
Insomnia	2 (1.3)	1 (1.2)	3 (2.0)	0	3 (1.2)
Skin and subcutaneous tissue disorders, *n* (%)	9 (5.7)	2 (2.4)	8 (5.4)	3 (4.0)	11 (4.5)
Decubitus ulcer	2 (1.3)	1 (1.2)	2 (1.3)	1 (1.3)	3 (1.2)
Rash	2 (1.3)	1 (1.2)	2 (1.3)	1 (1.3)	3 (1.2)
Ear and labyrinth disorders, *n* (%)	5 (3.2)	0	4 (2.7)	0	5 (2.1)
Ear pain	3 (1.9)	0	3 (2.0)	0	3 (1.2)

*Note*: Ambulatory status was defined as Gross Motor Function Classification System (GMFCS) levels I–III; non‐ambulatory status as GMFCS levels IV–V.

Abbreviations: SAE, serious adverse event; TEAE, treatment‐emergent adverse event.

Twenty‐five patients (10.3%) reported 48 serious adverse events (SAEs), of whom 13 patients (52.0% [13 out of 25] of patients with SAEs) were non‐ambulatory. The most common SAEs were seizure (1.7% of patients), pneumonia (1.2%), status epilepticus (1.2%), and tendinous contracture (1.2%). No SAEs were considered treatment‐related. Incidences of seizures as SAEs occurred in four patients, all of whom were aged 2 to 9 years, were previously treated, and had significant medical and surgical histories and three of whom had bilateral spasticity. Of the patients with seizure SAEs, one had a dual diagnosis of CP and epilepsy, one had a diagnosis of CP, one had a history of seizures, and one had a previous acute ischemic left middle cerebral artery stroke.

Sixty‐two patients (29.5%) from the effectiveness population withdrew from the study. From most to least, the primary reason for withdrawal was lost to follow‐up (*n =* 20, 32.3%), administrative reasons (*n =* 11, 17.7%), lack of efficacy (*n =* 8, 12.9%), surgery performed or planned (*n =* 8, 12.9%), and adverse events (*n =* 2, 3.2%). Adverse events leading to study discontinuation were deemed treatment‐related in only one patient who was receiving ancillary injections and whose symptoms consisted of generalized weakness for 1 month post‐injection of 1000 U in total to lower extremities. Most patients (*n =* 41 out of 62, 66.1%) who withdrew from the study were previously treated with a botulinum neurotoxin (BoNT).

## DISCUSSION

This prospective, observational study aimed to provide important information on the effectiveness, safety, and administration patterns of aboBoNT‐A for the treatment of PLLS in a real‐world setting. Chemodenervation with aboBoNT‐A improved the patients' ability to achieve their treatment goals, with results supporting the importance of an accurate and clear patient‐centered goal‐setting process during treatment. Some goals may have been more difficult to achieve, requiring several treatment cycles; however, the results corroborate that the selected goals were realistic and substantive. The results were found to be comparable between young children and adolescents and between BoNT‐naïve and previously treated patients, and were consistent overall with the results of the phase III clinical trial, showing that chemodenervation with aboBoNT‐A improves the ability of patients to achieve their treatment goals.^18^, ^19^ AboBoNT‐A doses were consistent with USPI^10^ across all cycles, and more than 80% of patients used concomitant non‐drug therapies (mainly physical/occupational therapy and cast application).

Ambulatory patients had a higher goal achievement rate than those who were non‐ambulatory. The process of setting and measuring appropriate goals can be challenging across different populations of patients.^12^, ^20^ Patients who are non‐ambulatory have limited physical function and independence,^21^ rely on several assistive devices and environmental modifications,^22^ and require a longer timeframe for goal achievement than patients who are ambulatory;^23^ in addition, caregiver‐selected goals are frequently aspirational and prioritize long‐term outcomes, which may further complicate timely goal attainment and contribute to psychological and motivational challenges. The percentage of patients who achieved their GAS T primary goal was 74.8% at the end of cycle 1 and was greater than 60% up to the end of cycle 8, reflecting sustained goal attainment. Consistent with these results, the percentage of participants who achieved GAS T primary goals overall (i.e. the number of achieved primary goals across all cycles exceeded the number of non‐achieved primary goals) was high. The observed increases in PedsQL total at cycles 8 and 9 and physical health summary scores at cycles 2 and 3 and stability across cycles further suggest that treatment with aboBoNT‐A has a favorable impact on quality of life. The study revealed no new safety concerns and confirmed that the safety profile of aboBoNT‐A in PLLS remains consistent with its established profile.

Treatment of children with spasticity with aboBoNT should be personalized as part of an integrative plan. While the USPI^10^ offers guidance on dosing, real‐world practice often differs owing to the variability in patients' presentations and the severity of spasticity, as evidenced by some of the ancillary total doses administered in this study being higher than the maximum recommended dose. Overall, the observed injection patterns will probably have been tailored to treatment goals being set forth by patients and the treatment team, which also depend on GMFCS level and quality of life impacts. This study provides real‐world total injection dose ranges specific to aboBoNT, which can be applied with clinical judgement or used as a reference point for clinicians when considering individualized treatment plans.

This study had several limitations. The absence of randomization and the 10‐patient limit per site may have resulted in selection bias, with the latter through underrepresentation of less common PLLS presentations. Study participation was voluntary for sites and patients; thus, volunteer bias cannot be ruled out, and participating sites may not be representative of all centers that manage and treat patients with PLLS. Missing data for different outcome measures may have caused an overestimation or underestimation of the results. Although the follow‐up was extended to 30 months after the first injection, fewer than 50% of those enrolled in the study had an injection after 18 months. Because of the observational nature of this study, disease assessment methods were not standardized across sites. The GMFCS, used at the inclusion visit to assess gross motor function, was developed for CP,^24^ whereas 17.4% of study participants had other types of acquired brain injury. Patients' cognitive or verbal skills were not assessed. Finally, the last injection cycle (cycle 10) had a limited number of patients with available data, thus results should be interpreted with caution. Despite these limitations, this study provides meaningful information about treatment with aboBoNT‐A and outcomes in the real‐life management of PLLS.

## CONCLUSION

This study provides insights into the effectiveness, safety, and patterns of the use of aboBoNT‐A to treat spasticity in children and adolescents with PLLS during a period of up to 30 months. The safety results are consistent with the known safety profile of aboBoNT‐A, with no new safety concerns identified for the management of PLLS. The effectiveness results further support chemodenervation with aboBoNT‐A as an effective treatment option with a positive risk–benefit profile for treating PLLS across patient disability levels, including non‐ambulatory patients. By capturing real‐world dosing trends, this observational study helps bridge the gap between clinical guidelines and actual practice.

## FUNDING INFORMATION

Ipsen (Cambridge, MA, USA).

## Conflict of Interest Statement

M.E. Gormley: Consultant: Ipsen; Research support: Ipsen; E. Dabrowski: Consultant: Allergan, Ipsen, Merz, Solstice; Research support: Ipsen, Merz; M.R. Delgado: Consultant: Allergan, Ipsen, and Kashiv Pharma; A. Tilton: Consultant: Ipsen; Research support: Ipsen; A. Christian: Research support: Ipsen; Employment: Good Shepherd Rehab Hospital; S.H. Evans: Consultant: Biogen, Ipsen; A.‐S. Grandoulier: Employment: Ipsen; J. Goldberg: Employment: Formerly of Ipsen.

## Supporting information


**Figure S1:** Patient disposition.

**Table S1:** Summary of Goal Attainment Scale for measurement of individual therapy goals.

**Table S2:** Most frequently selected goals (effectiveness population).

**Table S3:** Summary of primary goal achievement by end of each cycle (effectiveness population).

## Data Availability

Qualified researchers may request access to patient‐level study data that underlie the results reported in this publication. Additional relevant study documents, including the clinical study report, study protocol with any amendments, annotated case report form, statistical analysis plan, and dataset specifications may also be made available. Patient level data will be anonymized, and study documents will be redacted to protect the privacy of study participants. Where applicable, data from eligible studies are available 6 months after the studied medicine and indication have been approved in the US and EU or after the primary manuscript describing the results has been accepted for publication, whichever is later. Further details on Ipsen's sharing criteria, eligible studies, and process for sharing are available here (https://vivli.org/members/ourmembers/). Any requests should be submitted to www.vivli.org for assessment by an independent scientific review board.
