# Supplementary figures and images for: Effectiveness and safety of abobotulinumtoxinA in pediatric lower limb spasticity: A phase IV, prospective, observational, multicenter study
Source: Dev Med Child Neurol. 2025 Jul 31;68(2):227–39. doi: 10.1111/dmcn.16428 (PMC12766550; doi:10.1111/dmcn.16428)

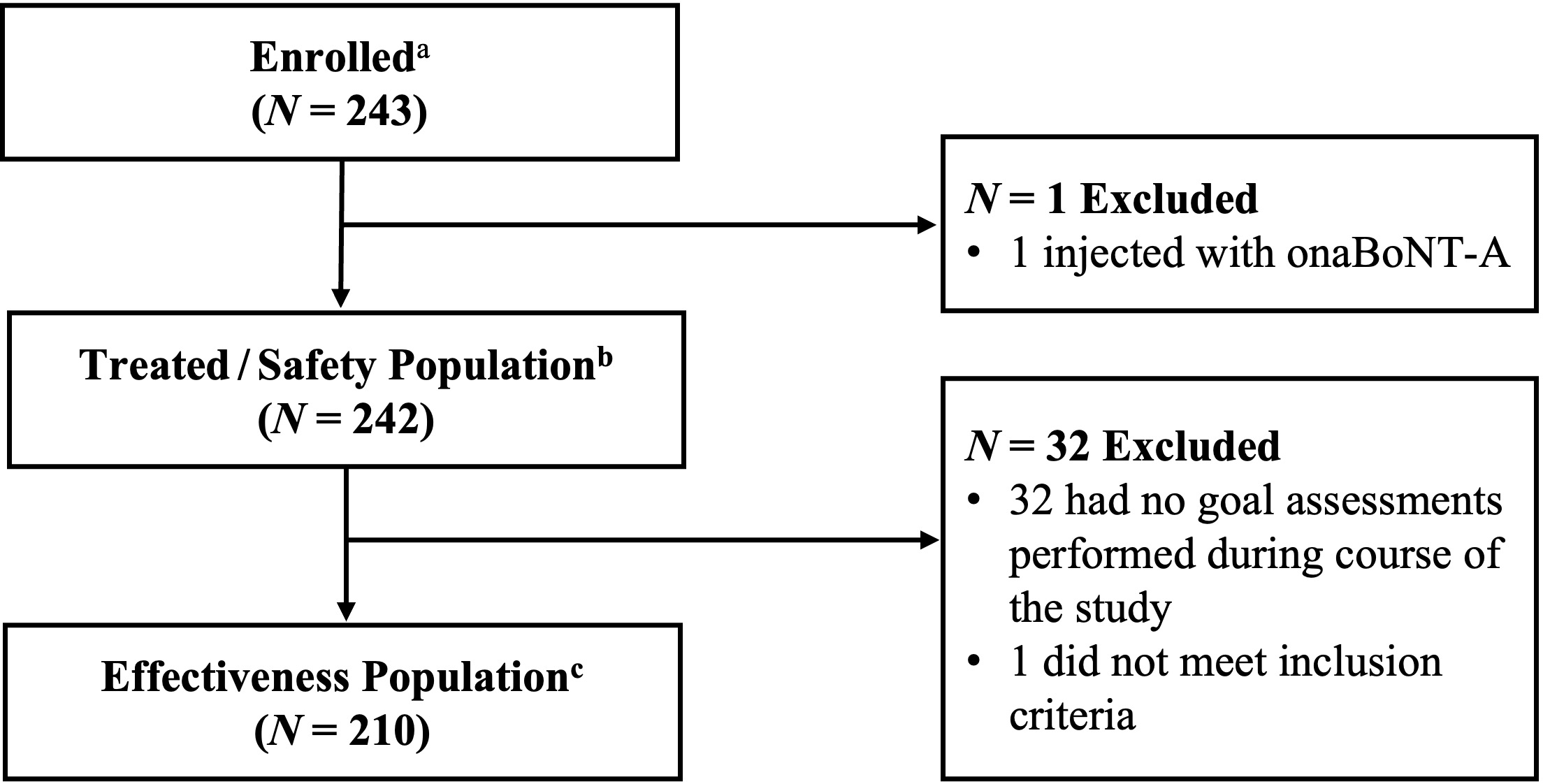

Supplement: Supplementary file 1 — Figure S1: Patient disposition. [file DMCN-68-227-s004.jpg]
