# Supplementary material for: Effectiveness and safety of abobotulinumtoxinA in pediatric lower limb spasticity: A phase IV, prospective, observational, multicenter study
Source: Dev Med Child Neurol. 2025 Jul 31;68(2):227–39. doi: 10.1111/dmcn.16428 (PMC12766550; doi:10.1111/dmcn.16428)
Supplement: Supplementary file 2 — Table S1: Summary of Goal Attainment Scale for measurement of individual therapy goals. [file DMCN-68-227-s001.docx]

**Table S1.** Summary of Goal Attainment Scale (GAS) for measurement of individual therapy goals

The GAS is a functional scale used to measure progress towards individual therapy goals.^1^ At each injection visit, individual goals were defined for each study participant by the physician and the child's parent/caregiver where applicable, prior to treatment. Goals chosen were to be realistic and achievable within 1 treatment cycle and based on the SMART (Specific, Measurable, Attainable, Relevant and Timely) principle from a pre-selected list below.

**Goal list:** Improved endurance; Looks better; Improved walking pattern; Increased walking speed; Improved balance; Decreased frequency of tripping; Decreased frequency of falling; Decreased foot pain; Longer shoe wear; Improved tolerance of the Ankle foot orthosis (AFO); Improved ease in putting on the AFO; Increased ease in performing activities of daily living; Improved hygiene; Improved positioning in wheelchair; Improved comfort, other (please

specify).

After goal selection, the study investigator then rated the level of difficulty of each chosen goal. Each goal was then scored using the 5-point scale below at each injection visit.

| −2 | Much less than expected outcome |
| --- | --- |
| −1 | Somewhat less than expected outcome |
| 0 | Expected outcome |
| +1 | Somewhat more than expected outcome |
| +2 | Much more than expected outcome |

Outcome data were then converted to a T-score which is normally distributed about a mean of 50 (if the goals are achieved precisely). A score greater than 50 represents a better than expected outcome.

^1^Turner-Stokes L. Goal attainment scaling (GAS) in rehabilitation: a practical guide. *Clin Rehabil*. 2009; 23(4):362-70.
