# Supplementary material for: Effectiveness and safety of abobotulinumtoxinA in pediatric lower limb spasticity: A phase IV, prospective, observational, multicenter study
Source: Dev Med Child Neurol. 2025 Jul 31;68(2):227–39. doi: 10.1111/dmcn.16428 (PMC12766550; doi:10.1111/dmcn.16428)
Supplement: Supplementary file 3 — Table S2: Most frequently selected goals (effectiveness population). [file DMCN-68-227-s003.docx]

**Table S2.** Most frequently selected goals (effectiveness population)

| **Goal selected** | ***n* (%)** |
| --- | --- |
| Improved walking patterns | 109 (51.9) |
| Improved balance | 59 (28.1) |
| Improved endurance | 52 (24.8) |
| Improved tolerance of ankle foot orthosis | 45 (21.4) |
| Decreased frequency of falling | 34 (16.2) |
| Decreased frequency of tripping | 27 (12.9) |
| Increased ease in performing activities of daily living | 27 (12.9) |
| Improved comfort | 22 (10.5) |
| Improved hygiene | 20 (9.5) |
| Improved ease in putting on ankle foot orthosis | 16 (7.6) |
| Increased walking speed | 16 (7.6) |
| Looks better | 13 (6.2) |
| Decreased foot pain | 12 (5.7) |
| Improved positioning in wheelchair | 9 (4.3) |
| Longer shoe wear | 4 (1.9) |
| Other | 97 (16.2) |
