# Supplementary material for: Effectiveness and safety of abobotulinumtoxinA in pediatric lower limb spasticity: A phase IV, prospective, observational, multicenter study
Source: Dev Med Child Neurol. 2025 Jul 31;68(2):227–39. doi: 10.1111/dmcn.16428 (PMC12766550; doi:10.1111/dmcn.16428)
Supplement: Supplementary file 4 — Table S3: Summary of primary goal achievement by end of each cycle (effectiveness population). [file DMCN-68-227-s002.docx]

**Table S3.** Summary of primary goal achievement by end of each cycle (effectiveness population)

|  | **Cycle 1**  *n/N* (%) | **Cycle 2**  *n/N* (%) | **Cycle 3**  *n/N* (%) | **Cycle 4**  *n/N* (%) | **Cycle 5**  *n/N* (%) | **Cycle 6**  *n/N* (%) | **Cycle 7**  *n/N* (%) | **Cycle 8**  *n/N* (%) | **Cycle 9**  *n/N* (%) |
| --- | --- | --- | --- | --- | --- | --- | --- | --- | --- |
| **Improved walking patterns** | 73/89 (82.0) | 41/63 (65.1) | 37/51 (72.5) | 27/38 (71.1) | 21/29 (72.4) | 12/19 (63.2) | 7/10 (70.0) | 2/4  (50.0) | 0/1  (0.0) |
| **Improved balance** | 21/33 (63.6) | 11/23 (47.8) | 15/21 (71.4) | 14/18 (77.8) | 8/12 (66.7) | 10/12 (83.3) | 4/8  (50.0) | 3/5  (60.0) | 1/3  (33.3) |
| **Improved endurance** | 19/31 (61.3) | 8/20 (40.0) | 8/15 (53.3) | 6/15 (40.0) | 8/13 (61.5) | 6/10 (60.0) | 3/4  (75.0) | 2/3  (66.7) | – |
| **Improved tolerance of ankle foot orthosis** | 20/29 (69.0) | 17/22 (77.3) | 9/14 (64.3) | 5/11 (45.5) | 2/4  (50.0) | 2/2  (100.0) | 1/2 (50.0) | 0/2 (0.0) | 0/2 (0.0) |
| **Decreased frequency of falling** | 17/24 (70.8) | 14/17 (82.4) | 10/13 (76.9) | 6/13 (46.2) | 7/8 (87.5) | 4/4 (100.0) | 3/3 (100.0) | 1/1 (100.0) | – |
| **Decreased frequency of tripping** | 16/17 (94.1) | 6/12 (50.0) | 9/10 (90.0) | 4/5  (80.0) | 2/2 (100.0) | 1/2 (50.0) | 2/2 (100.0) | 2/2 (100.0) | – |
| **Increased ease in performing activities of daily living** | 7/11 (63.6) | 7/8 (87.5) | 3/7 (42.9) | 8/10 (80.0) | 3/4 (75.0) | 1/2 (50.0) | 2/3 (66.7) | 1/2 (50.0) | 2/2 (100.0) |
| **Improved comfort** | 9/14 (64.3) | 6/6 (100.0) | 5/7 71.4) | 5/5 (100.0) | 4/4 (100.0) | 3/5 (60.0) | 3/4 (75.0) | 2/2 (100.0) | 1/1 (100.0) |
| **Improved hygiene** | 13/17 (76.5) | 5/6 (83.3) | 5/6 (83.3) | 1/2 (50.0) | 1/2 (50.0) | – | – | 1/1 (100.0) | – |
| **Improved ease in putting on ankle foot orthosis** | 6/7 (85.7) | 5/5 (100.0) | 6/6 (100.0) | 3/4 (75.0) | 2/2 (100.0) | – | – | 1/1 (100.0) | – |
| **Increased walking speed** | 4/7 (57.1) | 6/8 (75.0) | 3/3 (100.0) | 3/3 (100.0) | 3/3 (100.0) | – | – | – | – |
| **Looks better** | 6/10 (60.0) | 2/6  (33.3) | 1/4  (25.0) | 0/1 (0.0) | 1/1 (100.0) | – | – | – | – |
| **Decreased foot pain** | 5/5 (100.0) | 5/6 (83.3) | 4/4 (100.0) | 2/3 (66.7) | 1/1 (100.0) | – | – | 1/1 (100.0) | – |
| **Improved positioning in wheelchair** | 3/7 (42.9) | 4/6 (66.7) | 1/3 (33.3) | – | – | – | – | – | – |
| **Longer shoe wear** | 1/1 (100.0) | 1/1 (100.0) | 2/2 (100.0) | – | – | – | 1/1 (100.0) | – | – |
| **Other** | 61/78 (78.2) | 44/66 (66.7) | 37/55 (67.3) | 17/34 (34.1) | 20/41 (34.3) | 21/32 (65.6) | 6/12 (50.0) | 2/6  (33.3) | 0/1 (0.0) |

Percentages are based on the number of goals set (and assessed) per goal area and cycle.
